# Supplementary material for: Genetic correlation between amyotrophic lateral sclerosis and schizophrenia
Source: Nat Commun. 2017 Mar 21;8:14774. doi: 10.1038/ncomms14774 (PMC5364411; doi:10.1038/ncomms14774)
Supplement: Supplementary Information — Supplementary Figures, Supplementary Tables, Supplementary Notes, Supplementary Methods, and Supplementary References [file ncomms14774-s1.pdf]

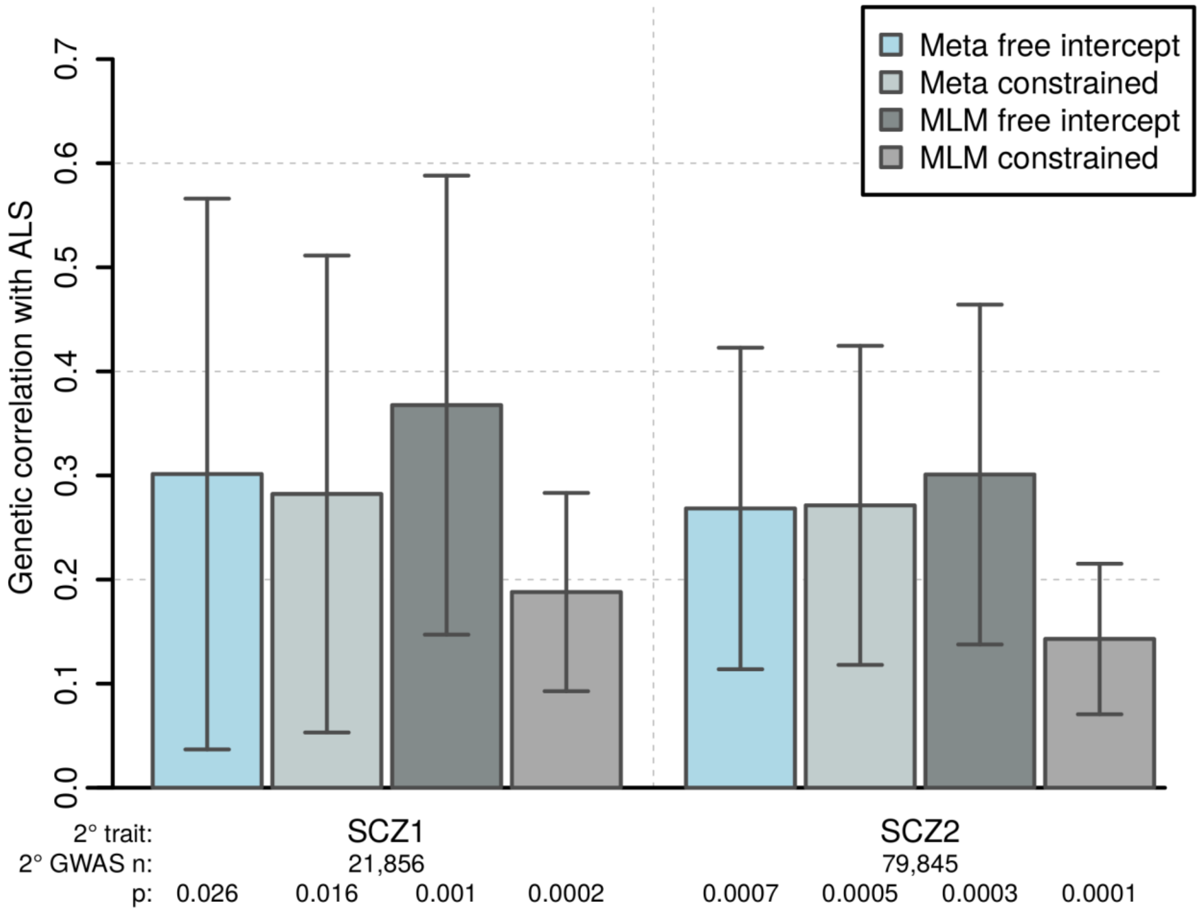

Supplementary figure 1 Comparison of genetic correlation estimates for two schizophrenia cohorts. SCZ1 denotes data from the 2011 study by the Schizophrenia Psychiatric Genome-Wide Association Study Consortium (European ancestry cohort); SCZ2 indicates data from the 2014 study by the Schizophrenia Working Group of the Psychiatric Genomics Consortium (European and Asian ancestry cohort). Error bars indicate 95% confidence intervals.

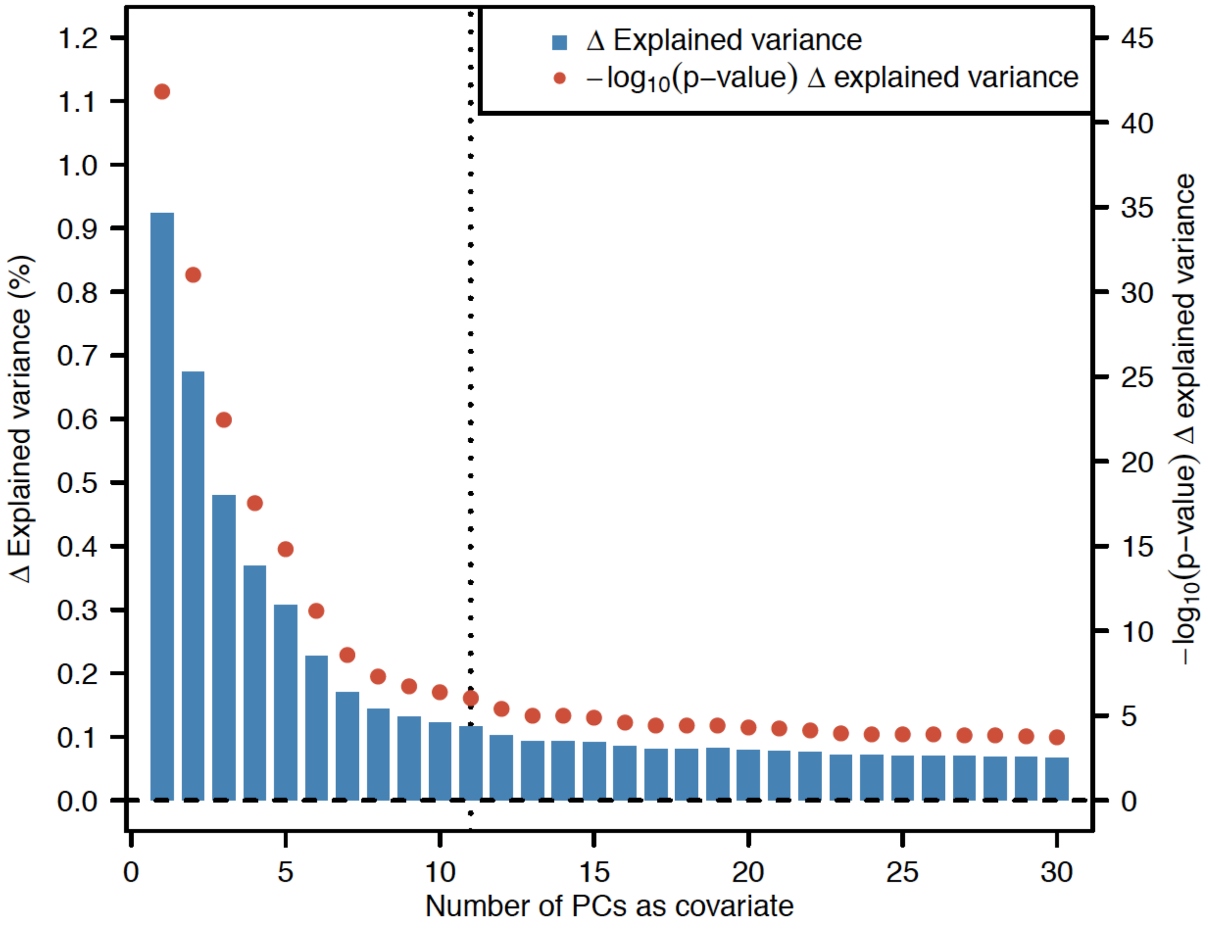

Supplementary figure 2 Effect of increasing the number of PCs as covariates in generalized linear model on explained variance in ALS and -log(p-values) attributable to polygenic risk scores (Δ explained variance) at schizophrenia P_T = 0.2. The dotted line indicates the 11 most significant PCs ($p < 0.0005$) used in the main analysis.

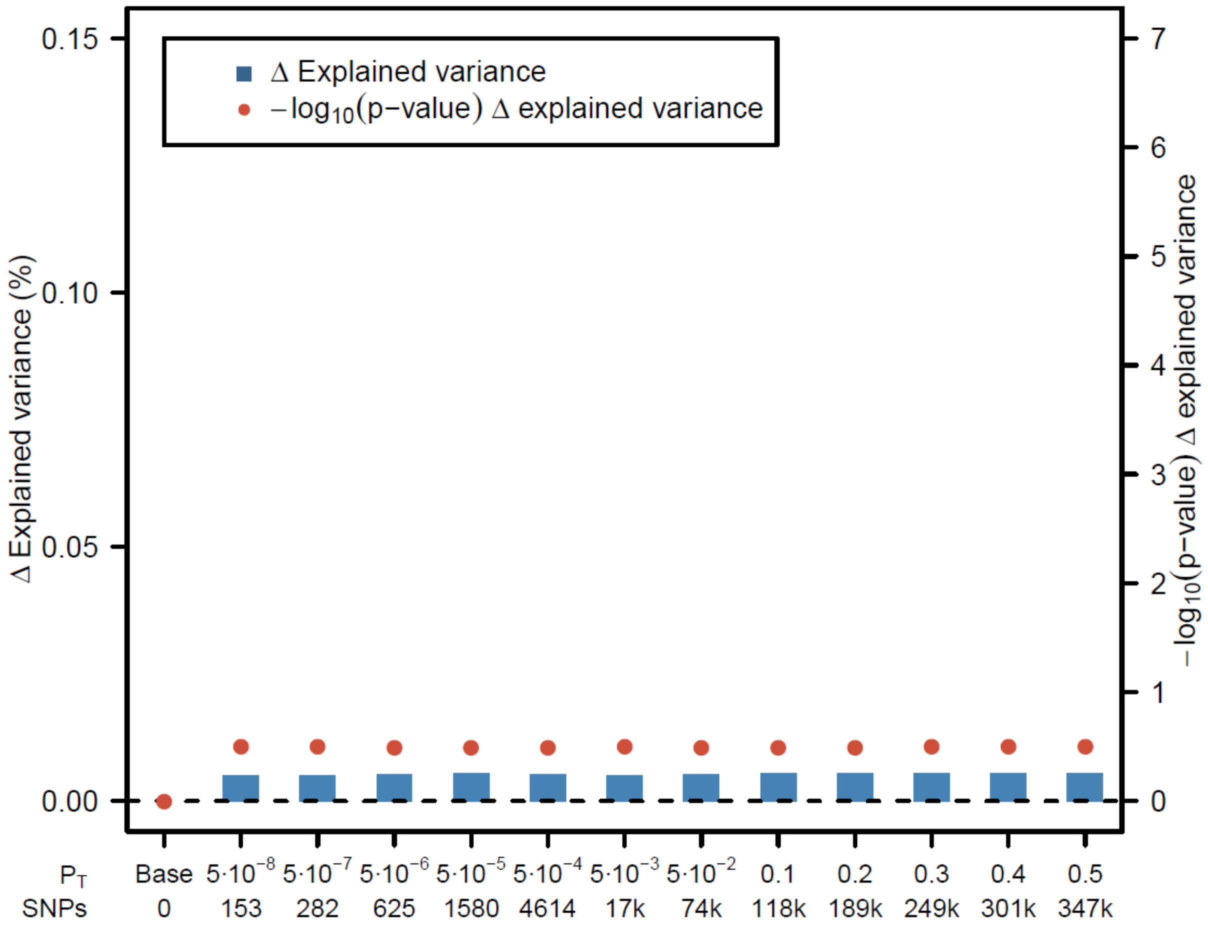

Supplementary figure 3 Effect of 1000 permutations of case-control labels, with conservation of case:control ratio, on analysis of polygenic risk scores (PRS) for schizophrenia in the ALS dataset. The schizophrenia P_T and associated number of SNPs are similar to the main analysis. Blue bars show the averaged **Δ** explained variance of a linear model containing PRS compared to a baseline model with only covariates. Red dots indicate averaged $-\log_{10}$ p-values of **Δ** explained variance.

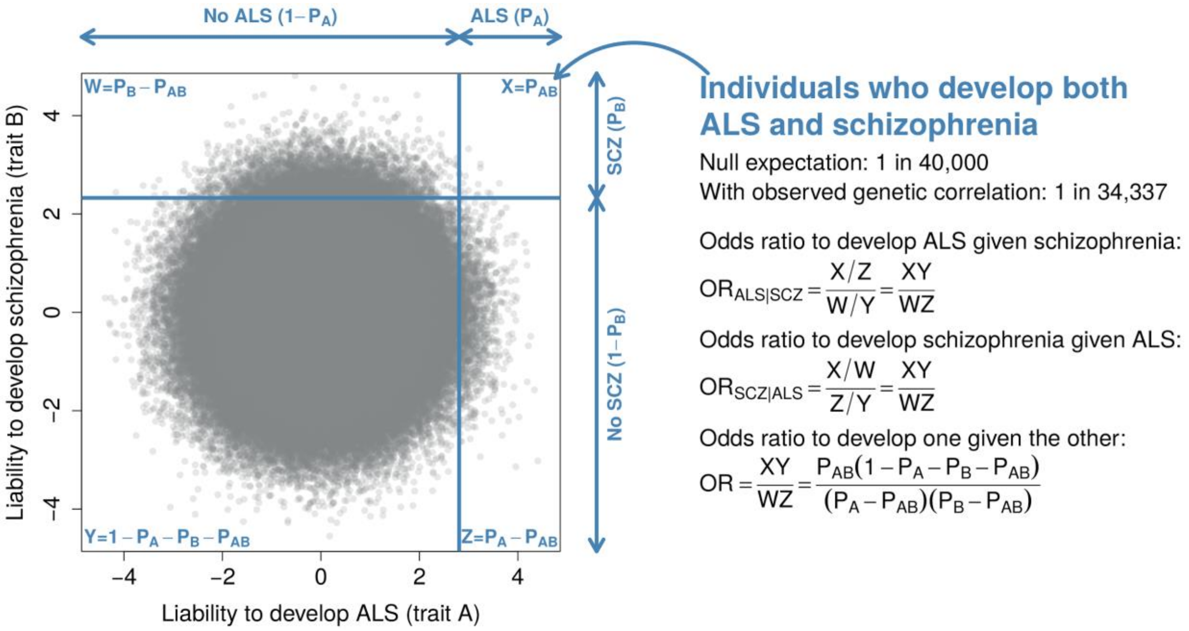

Supplementary figure 4 Simulated example of the bivariate distribution of liability for two traits with a genetic covariance (liability-scale) of 1.88% in a population of 500,000 individuals (grey dots). P_A and P_B represent the proportions of the population that carry above-threshold liabilities for traits A and B (defined by the lifetime risks for these traits). Individuals in sector X in the plot carry above-threshold liability for both traits A and B (P_{AB} of the population).

Supplementary table 1 LD score regression heritability estimates for amyotrophic lateral sclerosis

**Method**	**h_S^2 (%)**	**95% CI**
MLM constrained intercept	8.17	7.21-9.13
MLM free intercept	1.54	0.13-2.95
Meta constrained intercept	1.34	0.61-2.07
Meta free intercept	1.61	0.67-2.55
Permuted constrained intercept	-0.7	-1.50-0.18
Permuted free intercept	0.09	-1.09-1.27

h_S^2, SNP-based heritability; CI, confidence interval; MLM, mixed linear model summary statistics; meta, meta-analysis summary statistics

Supplementary table 2 Genetic correlation between amyotrophic lateral sclerosis and 8 secondary traits

	**2° Trait**	**AD**	**ADHD**	**ASD**	**BPD**	**HEIGHT**	**MDD**	**MS**	**SCZ**
	**GWAS n**	54,162	5,422	10,263	16,731	253,288	18,759	17,597	79,845
	K	0.15	0.05	0.01	0.01	n/a	0.15	0.0029	0.01
	**h_S^2 % (s.e.)**	8.1 (1.6)	9.5 (6.7)	17.0 (2.3)	17.1 (2.1)	27.4 (1.6)	13.4 (3.1)	19.1 (7.8)	19.7 (1.0)
MLM cons	**ρ_g % (s.e.)**	0.5 (0.8)	-0.7 (1.9)	-1.0 (1.0)	-0.2 (0.8)	-0.7 (0.4)	0.02 (1.0)	0.3 (0.6)	1.8 (0.5)
	**r_g % (s.e.)**	6.3 (9.6)	-8.3 (21.9)	-8.6 (8.7)	-1.9 (6.6)	-4.5 (2.9)	0.2 (9.8)	2.1 (4.7)	14.3 (3.7)
	**p**	0.51	0.71	0.32	0.78	0.11	0.98	0.65	**0.0001**
MLM free	**ρ_g % (s.e.)**	1.5 (1.8)	-1.6 (4.0)	-3.1 (2.2)	-0.3 (1.6)	-1.4 (1.0)	-0.3 (1.9)	0.9 (1.4)	3.8 (1.1)
	**r_g % (s.e.)**	18.8 (22.4)	-18.4 (44.7)	-25.8 (19.0)	-2.9 (13.4)	-9.2 (6.7)	-2.8 (18.3)	6.9 (11.0)	30.1 (8.3)
	**p**	0.40	0.68	0.18	0.83	0.18	0.88	0.53	**0.0003**
Meta cons	**ρ_g % (s.e.)**	1.9 (1.8)	-0.9 (3.2)	-2.3 (2.1)	-0.9 (1.5)	-0.2 (0.9)	0.1 (2.0)	1.0 (1.2)	3.4 (1.0)
	**r_g % (s.e.)**	23.3 (21.8)	-10.0 (36.3)	-19.1 (18.0)	-8.0 (12.6)	-1.3 (6.0)	1.3 (18.9)	8.3 (9.8)	27.1 (7.8)
	**p**	0.28	0.78	0.29	0.52	0.83	0.94	0.40	**0.0005**
Meta free	**ρ_g % (s.e.)**	2.0 (1.8)	-0.9 (3.6)	-2.4 (2.2)	-1.1 (1.7)	-0.2 (0.9)	0.1 (2.2)	1.2 (1.3)	3.4 (1.0)
	**r_g % (s.e.)**	24.5 (21.6)	-10.4 (40.5)	-20.1 (19.0)	-9.1 (14.2)	-1.4 (6.3)	0.9 (21.1)	9.2 (10.5)	26.8 (7.9)
	**p**	0.26	0.80	0.27	0.52	0.83	0.97	0.38	**0.0007**

AD, Alzheimer's disease; ADHD, attention deficit-hyperactivity disorder; ASD, autism spectrum disorder; BPD, bipolar disorder; MDD, major depressive disorder; MS, multiple sclerosis; SCZ, schizophrenia; n, sample size; K, population prevalence (lifetime risk) for binary traits; h_S^2, SNP-based heritability; ρ_g, genetic covariance; r_g, genetic correlation; s.e., standard error; MLM, mixed linear model summary statistics for ALS; Meta, meta-analysis summary statistics for ALS; cons, constrained-intercept LD score regression for primary trait (ALS) h_S^2 estimation

p-values are emboldened if they are significant at (conservative) Bonferroni-corrected α = 0.0016

h_S^2 and ρ_g estimates are liability-scale for binary traits

h_S^2 estimates for secondary traits used free-intercept LD score regression

Supplementary table 3 Number of individuals in the ALS dataset excluded from PRS analysis due to (suspected) sample overlap with the schizophrenia dataset

Stratum	Name	Total individuals	Excluded individuals	Percentage
**1**	NL1	843	5	0.6
**2**	BE1	616	1	0.2
**3**	NL2	5027	25	0.5
**4**	SW1	556	82	14.7
**5**	FR1	809	0	0.0
**6**	UK1	327	0	0.0
**7**	US1	1937	4	0.2
**8**	IR1	639	16	2.5
**9**	UK2	3301	2555	77.4
**10**	US2	779	4	0.5
**11**	IT1	626	0	0.0
**12**	FIN1	756	756	100.0
**13**	NL3	2781	86	3.1
**14**	GER1	776	776	100.0
**15**	IT2	383	0	0.0
**16**	IB1	225	0	0.0
**17**	SWISS1	424	0	0.0
**18**	BE2	447	1	0.2
**19**	SW2	467	60	12.8
**20**	FIN2	232	232	100.0
**21**	IR2	707	299	42.3
**22**	US3	2562	3	0.1
**23**	FR2	1332	0	0.0
**24**	UK3	3534	2433	68.8
**25**	GER2	2047	2047	100.0
**26**	IT3	2790	0	0.0
**27**	NL4	1129	8	0.7
	**Total**	**36052**	**9393**	**26.1**

NL, Netherlands; BE, Belgium; SW, Sweden; FR, France; UK, United Kingdom; US; United States of America; IR, Ireland; IT, Italy; FIN, Finland; GER, Germany; IB, Iberia (Spain and Portugal); SWISS, Switzerland.

Supplementary table 4 Genomic regions with high LD removed prior to polygenic risk score calculation

Chromosome	Basepair start	Basepair end
**1**	48000000	52000000
**2**	86000000	100500000
	183000000	190000000
**3**	47500000	50000000
	83500000	87000000
**5**	44500000	50500000
	129000000	132000000
**6**	25500000	33500000
	57000000	64000000
	140000000	142500000
**7**	55000000	66000000
**8**	8000000	12000000
	43000000	50000000
	8135000	12000000
	112000000	115000000
**10**	37000000	43000000
**11**	87500000	90500000
**12**	33000000	40000000
**17**	40900000	45000000
**20**	32000000	34500000

Positions refer to human genome build GRCh37

Supplementary table 5 Estimated explained variances and p-values of polygenic risk scores through generalized linear model

SCZ P_T	n SNPs	Explained variance (Nagelkerke R^2, %)	Δ Explained variance (Nagelkerke R^2, %)	p-value	$-\log_{10}$(p-value)
**Baseline**	0	9.050	-	-	-
**$5×10^{-8}$**	153	9.051	0.001	6.90E-01	0.16
**$5×10^{-7}$**	282	9.056	0.006	2.73E-01	0.56
**$5×10^{-6}$**	625	9.053	0.002	5.10E-01	0.29
**$5×10^{-5}$**	1,580	9.057	0.007	2.36E-01	0.63
**$5×10^{-4}$**	4,614	9.081	0.030	1.15E-02	1.94
**$5×10^{-3}$**	17,042	9.126	0.075	7.15E-05	4.15
**0.05**	74,036	9.143	0.093	1.01E-05	4.99
**0.1**	118,335	9.163	0.112	1.27E-06	5.90
**0.2**	189,492	9.166	0.116	8.43E-07	6.07
**0.3**	249,452	9.162	0.111	1.41E-06	5.85
**0.4**	301,405	9.164	0.114	1·06E-06	5.97
**0.5**	346,683	9.161	0.111	1·50E-06	5.82

SCZ, schizophrenia; P_T, p-value threshold

Supplementary table 6 ALS case-control ratio for schizophrenia polygenic risk score deciles

Decile	Cases	Controls	OR vs decile 1	95% CI	Z value	p-value
**1**	732	1,934	-	-	-	-
**2**	801	1,865	1.02	0.90-1.15	0.27	$7.6×10^{-1}$
**3**	882	1,784	1.11	0.98-1.25	1.60	$9.4×10^{-2}$
**4**	910	1,756	1.10	0.97-1.24	1.47	$1.3×10^{-1}$
**5**	967	1,698	1.13	1.00-1.27	1.89	$4.8×10^{-2}$
**6**	982	1,685	1.10	0.97-1.24	1.39	$1.3×10^{-1}$
**7**	1,056	1,610	1.16	1.03-1.31	2.26	$1.7×10^{-2}$
**8**	1,103	1,562	1.15	1.02-1.30	2.06	$2.6×10^{-2}$
**9**	1,224	1,442	1.25	1.10-1.41	3.35	$3.9×10^{-4}$
**10**	1,375	1,291	1.30	1.15-1.48	3.84	$5.1×10^{-5}$

OR, odds ratio; CI, confidence interval

Supplementary table 7 Misdiagnosis rates (M_{TA}) of ALS as schizophrenia required to yield genetic correlation estimates assuming true genetic correlation of 0

Method	r_g (%)	95% CI	$\rho_{g,l}$ (%)	95% CI	M_{TA} (%)	95% CI
**MLM constrained**	14.3	7.08-21.6	1.88	0.92-2.83	4.86	2.47-7.13
**MLM free**	30.1	13.8-46.4	3.95	1.81-6.09	9.70	4.70-14.2
**Meta constrained**	27.1	11.8-42.4	3.55	1.55-5.56	8.82	4.04-13.1
**Meta free**	26.8	11.4-42.2	3.52	1.50-5.53	8.73	3.91-13.1

r_g, genetic correlation between ALS and schizophrenia; CI, confidence interval; $\rho_{g,l}$, liability-scale genetic covariance; M_{TA}, misdiagnosis rate

Supplementary table 8 Genomic loci associated with ALS at $\mathrm{cFDR}_{\mathrm{ALS|SCZ}} < 0.05$

Index SNP	Chromosome	Position (GRCh37)	GWAS p_{ALS}	GWAS p_{SCZ}	$\mathrm{cFDR}_{\mathrm{ALS|SCZ}}$	Closest gene
rs63535762	1	233663807	4.0882×10^{-6}	0.1232	0.02914227	*KCNK1*
rs11124852	2	42131864	5.6779×10^{-6}	0.04953	0.03113055	*C2orf91*
rs149853584	3	1728150	3.5418×10^{-8}	0.03095	0.00038322	*CNTN6*
rs2625891	3	9541761	2.763×10^{-6}	0.03099	0.01459386	*LHFPL4*
rs616147	3	39534481	5.7276×10^{-8}	0.6211	0.00133118	*MOBP*
rs1817362	3	147770849	8.3621×10^{-6}	0.009266	0.03495604	*AGTR1*
rs71308531	3	171681860	2.0263×10^{-6}	0.5228	0.02195072	*FDNC3B*
rs13164762	5	83141157	4.5304×10^{-6}	0.07264	0.02709394	*EDIL3*
rs34384833	5	91238155	9.9144×10^{-6}	0.0399	0.04847741	*ARRDC3*
rs189841212	5	117878641	6.5395×10^{-6}	0.02757	0.02927102	*DTWD2*
rs10463311	5	150410835	3.645×10^{-7}	0.1378	0.00317718	*TNIP1*
rs116946806	7	131682571	2.5608×10^{-6}	0.3095	0.02134998	*PLXNA4*
rs17070492	8	2420855	4.0231×10^{-6}	0.01049	0.01994008	*MYOM2*
rs7814166	8	143370923	6.8943×10^{-5}	1.373×10^{-10}	0.0427745	*TSNARE1*
rs10965525	9	22936164	5.0503×10^{-6}	0.006832	0.02977711	*DMRTA1*
rs2484319	9	27563755	2.0583×10^{-17}	0.007078	2.8215×10^{-12}	*C9orf72*
rs76794160	9	71621214	1.5665×10^{-6}	0.1176	0.01059827	*PIP5K1B*
rs11146348	10	133772553	7.1712×10^{-7}	0.0113	0.00453269	*PPP2R2D*
rs143276647	11	118299359	8.2063×10^{-6}	0.06059	0.03712072	*KMT2A*
rs79676202	12	50180558	4.5475×10^{-8}	0.369	0.00070155	*NCKAP5L*
rs113247976	12	57975700	4.1051×10^{-6}	0.1964	0.03831843	*KIF5A*
rs116900480	12	58656105	1.4775×10^{-6}	0.1303	0.01195632	*XRCC6BP1*
rs76805704	12	64532377	1.5297×10^{-6}	0.1331	0.01220552	*SRGAP1*
rs74654358	12	64881967	1.5956×10^{-7}	0.02022	0.00103714	*TBK1*
rs179552	14	31225831	9.8359×10^{-7}	0.1131	0.01156173	*SCFD1*
rs3097439	15	27871229	7.5601×10^{-6}	0.1372	0.0401236	*GABRG3*
rs35714695	17	26719788	3.1748×10^{-9}	0.7655	0.00023563	*SARM1*
rs78549703	19	17749542	1.6827×10^{-9}	0.1015	1.4414×10^{-5}	*UNC13A*
rs6015322	20	57206540	1.3812×10^{-6}	0.4992	0.03610187	*APCDD1L-AS1*
rs117635456	21	43460912	4.377×10^{-7}	0.3587	0.00546115	*ZNF295-AS1*
rs2176039	22	45585032	9.1138×10^{-6}	0.02482	0.04064275	*NUP50*

SNP, single nucleotide polymorphism; GWAS, genome-wide association study; ALS, amyotrophic lateral sclerosis; SCZ, schizophrenia; cFDR, conditional false discovery rate

Supplementary note 1: IGAP data

This study made use of data from the International Genomics of Alzheimer's Project (IGAP), a large two-stage study based upon genome-wide association studies (GWAS) on individuals of European ancestry. In stage 1, IGAP used genotyped and imputed data on 7,055,881 single nucleotide polymorphisms (SNPs) to meta-analyse four previously-published GWAS datasets consisting of 17,008 Alzheimer's disease cases and 37,154 controls (The European Alzheimer's disease Initiative – EADI the Alzheimer Disease Genetics Consortium – ADGC The Cohorts for Heart and Aging Research in Genomic Epidemiology consortium – CHARGE The Genetic and Environmental Risk in AD consortium – GERAD). In stage 2, 11,632 SNPs were genotyped and tested for association in an independent set of 8,572 Alzheimer's disease cases and 11,312 controls. Finally, a meta-analysis was performed combining results from stages 1 & 2.

Supplementary note 2: Consortium members

PARALS registry

Stefania Cammarosano[72], Antonio Canosa[72], Dario Cocito[297], Leonardo Lopiano[297], Luca Durelli[297], Bruno Ferrero[297], Antonio Bertolotto[298], Alessandro Mauro[297], Luca Pradotto[16], Roberto Cantello[299], Enrica Bersano[299,70], Dario Giobbe[297], Maurizio Gionco[300], Daniela Leotta[301], Lucia Appendino[302], Roberto Cavallo[303], Enrico Odddenino[304], Claudio Geda[305], Fabio Poglio[306], Paola Santimaria[307], Umberto Massazza[308], Angelo Villani[309], Roberto Conti[309], Fabrizio Pisano[310], Mario Palermo[311], Franco Vergnano[312], Paolo Provera[313], Maria Teresa Penza[314], Marco Aguggia[315], Nicoletta Di Vito[315], Piero Meineri[316], Ilaria Pastore[316], Paolo Ghiglione[317], Danilo Seliak[317], Nicola Launaro[318], Giovanni Astegiano[319], Bottacchi Edo[320]

SLALOM group

Francesca Gerardi[321], Fabrizio Rinaldi[322], Maria Sofia Cotelli[78], Luca Chiveri[323], Maria Cristina Guaita[324], Patrizia Perrone[324]

SLAP registry

Isabella Laura Simone[81], Stefano Zoccolella[81], Michele Zarrelli[325], Franco Apollo[325]

FALS Sequencing Consortium

Bradley N. Smith[8], Nicola Ticozzi[16,17], Claudia Fallini[85], Athina Soragia Gkazi[8], Simon Topp[8], Jason Kost[85], Emma L. Scotter[326], Kevin P. Kenna[85], Pamela Keagle[85], Jack W. Miller[8], Cinzia Tiloca[16], Caroline Vance[8], Eric W. Danielson[85], Claire Troakes[8], Claudia Colombrita[16,17], Safa Al-Sarraj[8], Elizabeth A. Lewis[327], Andrew King[8], Daniela Calini[16], Viviana Pensato[71], Barbara Castellotti[71], Jacqueline de Belleroche[328], Frank Baas[329], Anneloor L.M.A. ten Asbroek[329], Peter C. Sapp[85], Diane McKenna-Yasek[85], Russell L. McLaughlin[1,2], Meraida Polak[24], Seneshaw Asress[24], Jesús Esteban-Pérez[330,331], José Luis Muñoz-Blanco[332], Sandra D'Alfonso[68], Letizia Mazzini[70], Giacomo P. Comi[67], Roberto Del Bo[67], Mauro Ceroni[333], Stella Gagliardi[66], Giorgia Querin[69], Cinzia Bertolin[69], Wouter van Rheenen[3], Frank P. Diekstra[3], Rosa Rademakers[334], Marka van Blitterswijk[334], Kevin B. Boylan[335], Giuseppe Lauria[336], Stefano Duga[337], Stefania Corti[17], Cristina Cereda[66], Lucia Corrado[68,69], Gianni Sorarù[69], Kelly L. Williams[10], Garth A.

Nicholson[10,11], Ian P. Blair[10], Claire Leblond-Manry[338], Guy A. Rouleau[338], Orla Hardiman[1], Karen E. Morrison[27,28], Jan H. Veldink[3], Leonard H. van den Berg[3], Ammar Al-Chalabi[8], Hardev Pall[339,28], Pamela J. Shaw[29], Martin R. Turner[340], Kevin Talbot[340], Franco Taroni[71], Alberto García-Redondo[330,331], Zheyang Wu[341], Jonathan D. Glass[24], Cinzia Gellera[71], Antonia Ratti[16,17], Robert H. Brown Jr.87, Vincenzo Silani[16,17], Christopher E. Shaw[8], John E. Landers[90]

SLAGEN Consortium

Daniela Calini[16], Isabella Fogh[8,16], Antonia Ratti[16,17], Vincenzo Silani[16,17], Nicola Ticozzi[16,17], Cinzia Tiloca[16], Barbara Castellotti[71], Cinzia Gellera[71], Viviana Pensato[71], Franco Taroni[71], Cristina Cereda[66], Mauro Ceroni[333], Stella Gagliardi[66], Giacomo Comi[67], Stefania Corti[17], Roberto Del Bo[67], Lucia Corrado[68,69], Sandra D'Alfonso[68,69], Letizia Mazzini[70], Elena Pegoraro[69], Giorgia Querin[69], Gianni Sorarù[69]

NNIPPS Study Group

William Camu[342], Jean Sebastien Hulot[343], Francois Viallet[344], Philippe Couratier[345], David Maltete[346], Christine Tranchant[347], Marie Vidailhet[348]

Psychosis Endophenotypes International Consortium

Maria J Arranz[240,349], Steven Bakker[4], Stephan Bender[350,351], Elvira Bramon[240,352,353], David A Collier[101,102], Benedicto Crespo-Facorro[354,355], Jeremy Hall[218], Conrad Iyegbe[240], Assen V Jablensky[356], René S Kahn[4], Luba Kalaydjieva[186,357], Stephen Lawrie[218], Cathryn M Lewis[96,97], Kuang Lin[8], Don H Linszen[358], Ignacio Mata[354,355], Andrew M McIntosh[218], Robin M Murray[226], Roel A Ophoff[6], Jim Van Os[227,240], John Powell[8], Dan Rujescu[168,170], Muriel Walshe[240], Matthias Weisbrod[351], Durk Wiersma[359]

Wellcome Trust Case-control Consortium

Management Committee: Peter Donnelly[263,360], Ines Barroso[361], Jenefer M Blackwell[362,363], Elvira Bramon[59], Matthew A Brown[364], Juan P Casas[365,366], Aiden Corvin[98], Panos Deloukas[361], Audrey Duncanson[367], Janusz Jankowski[368], Hugh S Markus[369], Christopher G Mathew[370], Colin NA Palmer[371], Robert Plomin[102], Anna Rautanen[263], Stephen J Sawcer[372], Richard C Trembath[370], Ananth C Viswanathan[373,374], Nicholas W Wood[375]

Data and Analysis Group: Chris CA Spencer[263], Gavin Band[263], Céline Bellenguez[263], Peter Donnelly[263,360], Colin Freeman[263], Eleni Giannoulatou[263], Garrett Hellenthal[263], Richard Pearson[263], Matti Pirinen[263], Amy Strange[263], Zhan Su[263], Damjan Vukcevic[263]

DNA, Genotyping, Data QC and Informatics: Cordelia Langford[361], Ines Barroso[361], Hannah Blackburn[361], Suzannah J Bumpstead[361], Panos Deloukas[361], Serge Dronov[361], Sarah Edkins[361], Matthew Gillman[361], Emma Gray[361], Rhian Gwilliam[361], Naomi Hammond[361], Sarah E Hunt[361], Alagurevathi Jayakumar[361], Jennifer Liddle[361], Owen T McCann[361], Simon C Potter[361], Radhi Ravindrarajah[361], Michelle Ricketts[361], Avazeh Tashakkori-Ghanbaria[361], Matthew Waller[361], Paul Weston[361], Pamela Whittaker[361], Sara Widaa[361]

Publications Committee: Christopher G Mathew[370], Jenefer M Blackwell[362,363], Matthew A Brown[364], Aiden Corvin[98], Mark I McCarthy[376], Chris CA Spencer[263]

297 Department of Neuroscience, Ospedale Molinette, Turin, Italy
298 Division of Neurology, University Hospital San Luigi Gonzaga, Orbassano, Turin.
299 Department of Neurology, ALS Center, Azienda Ospedaliero Universitaria Maggiore della Carità, Novara, Italy
300 Department of Neurology, ‘Amedeo Avogadro’ University, Novara
301 Martini Hospital, Alzheimer Assessment Unit, UVA and Neurology Unit, Turin, Italy
302 Department of Neurology, Ospedale Maria Vittoria, Torino
303 Department of Neurology, Ospedale S. Giovanni Bosco, Torino
304 Department of Neurology, Ospedale Gradenigo, Torino
305 Department of Neurology, Ospedale di Ivrea
306 Department of Neurology, Ospedale di Pinerolo
307 Department of Neurology, Ospedale di Vercelli
308 Department of Neurology, Ospedale di Biella
309 Department of Neurology, Ospedale di Domodossola
310 Salvatore Maugeri Foundation, IRCSS, Scientific Institute of Veruno (NO), Veruno, Italy
311 Department of Neurology, Azienda Ospedaliera Santi Antonio e Biagio, Alessandria
312 Department of Neurology, Ospedale di Casale Monferrato
313 Department of Neurology, Ospedale di Novi Ligure
314 Department of Neurology, Tortona
315 Department of Neurology, Ospedale di Asti
316 Department of Neurology, Azienda Ospedaliera Santa Croce e Carle, Cuneo
317 Department of Neurology, Ospedale di Savigliano
318 Department of Anestesiology, Ospedale di Saluzzo
319 Department of Neurology, Ospedale di Alba
320 Department of Neurology, Azienda Ospedaliera Regionale di Aosta, Azienda USL Valle d'Aosta, Aosta, Italy
321 NEuroMuscular Omnicentre (NEMO), Fondazione Serena Onlus, Milan, Italy
322 Neurology Unit, Department of Clinical and Experimental Sciences, University of Brescia
323 “Valduce” General Hospital, Como
324 Legnano General Hospital, Legnano
325 U.O. di Neurologia Ospedale ‘Casa Sollievo della Sofferenza', IRCCS San Giovanni Rotondo, Foggia
326 Centre for Brain Research, University of Auckland, Auckland, New Zealand
327 Neurobiology Department, Aaron Lazare Research Building, University of Massachusetts Medical School, Worcester, MA 01605, USA
328 Neurogenetics Group, Division of Brain Sciences, Imperial College London, London, UK.
329 Department of Clinical Genetics, Academic Medical Centre, University of Amsterdam, Amsterdam, the Netherlands.
330 Unidad de ELA, Instituto de Investigación Hospital 12 de Octubre de Madrid, Madrid, Spain.
331 Centro de Investigación Biomédica en Red de Enfermedades Raras (CIBERER) U-723, Madrid, Spain.
332 Unidad de ELA, Instituto de Investigación Hospital Gregorio Marañón de Madrid, Madrid, Spain.

333 Department of Neurological Science, IRCCS "Istituto Nazionale Neurologico C. Mondino" Foundation, University of Pavia, Pavia, Italy
334 Department of Neuroscience, Mayo Clinic, Jacksonville, Florida, USA.
335 Department of Neurology, Mayo Clinic Florida, Jacksonville, Florida, USA.
336 3rd Neurology Unit, Motor Neuron Diseases Center, Fondazione IRCCS Istituto Neurologico 'Carlo Besta', Milan, Italy.
337 Department of Biomedical Sciences, Humanitas University, Rozzano, Milan, Italy
338 Montreal Neurological Institute, Department of Neurology and Neurosurgery, McGill University, Montreal, Quebec, Canada.
339 Institute of Clinical Studies, College of Medical and Dental Sciences, University of Birmingham, Edgbaston, Birmingham, UK.
340 Nuffield Department of Clinical Neurosciences, University of Oxford, Oxford, UK.
341 Department of Mathematical Sciences, Worcester Polytechnic Institute, 100 Institute Road, Worcester, MA 01609-2280, USA
342 Department of Neurology, CHU Montpellier, Hopital Guy de Chauliac, Montpellier, France
343 Sorbonne Universités, UPMC Univ Paris 06, Institute of Cardiometabolism and Nutrition (ICAN), Pitié-Salpêtrière Hospital, Paris F-75013, France
344 Department of Neurology, University Hospital, Avenue des Tamaris, 13616, Aix-En-Provence, France
345 CHU Limoges, Centre de Compétence SLA, Limoges, France
346 Department of Neurology, Rouen University Hospital and University of Rouen, France
347 CHU de Strasbourg-Hôpital de Hautepierre, Service de Neurologie, 67098, Strasbourg, France
348 Hôpital Universitaire Pitié-Salpêtrière, Sorbonne Universités, Paris, France
349 Fundació de Docència i Recerca Mútua de Terrassa, Universitat de Barcelona, Spain.
350 Child and Adolescent Psychiatry, University of Technology Dresden, Dresden, Germany.
351 Section for Experimental Psychopathology, General Psychiatry, Heidelberg, Germany.
352 Institute of Cognitive Neuroscience, University College London, London, UK.
353 Mental Health Sciences Unit, University College London, London, UK.
354 Centro Investigación Biomédica en Red Salud Mental, Madrid, Spain.
355 University Hospital Marqués de Valdecilla, Instituto de Formación e Investigación Marqués de Valdecilla, University of Cantabria, Santander, Spain.
356 Centre for Clinical Research in Neuropsychiatry, The University of Western Australia, Perth, Western Australia, Australia.
357 Western Australian Institute for Medical Research, The University of Western Australia, Perth, Western Australia, Australia.
358 Department of Psychiatry, Academic Medical Center, University of Amsterdam, Amsterdam, The Netherlands.
359 Department of Psychiatry, University Medical Center Groningen, University of Groningen, The Netherlands.
360 Department of Statistics, University of Oxford, Oxford, UK.
361 Wellcome Trust Sanger Institute, Wellcome Trust Genome Campus, Hinxton, Cambridge, UK.
362 Cambridge Institute for Medical Research, University of Cambridge School of Clinical Medicine, Cambridge, UK.

363 Telethon Institute for Child Health Research, Centre for Child Health Research, University of Western Australia, Subiaco, Western Australia, Australia.
364 Diamantina Institute of Cancer, Immunology and Metabolic Medicine, Princess Alexandra Hospital, University of Queensland, Brisbane, Queensland, Australia.
365 Department of Epidemiology and Population Health, London School of Hygiene and Tropical Medicine, London, UK.
366 Department of Epidemiology and Public Health, University College London, London, UK.
367 Molecular and Physiological Sciences, The Wellcome Trust, London, UK.
368 Peninsula School of Medicine and Dentistry, Plymouth University, Plymouth, UK.
369 Clinical Neurosciences, St George's University of London, London, UK.
370 Department of Medical and Molecular Genetics, School of Medicine, King's College London, Guy's Hospital, London, UK.
371 Biomedical Research Centre, Ninewells Hospital and Medical School, Dundee, UK.
372 Department of Clinical Neurosciences, University of Cambridge, Addenbrooke's Hospital, Cambridge, UK.
373 Institute of Ophthalmology, University College London, London, UK.
374 National Institute for Health Research, Biomedical Research Centre at Moorfields Eye Hospital, National Health Service Foundation Trust, London, UK.
375 Department of Molecular Neuroscience, Institute of Neurology, London, UK.
376 Oxford Centre for Diabetes, Endocrinology and Metabolism, Churchill Hospital, Oxford, UK.

Supplementary methods 1: estimation of diagnostic misclassification

A positive genetic correlation between ALS and schizophrenia could derive from misdiagnosis of cognitive- or behavioural-onset FTD-ALS as schizophrenia. For traits A and B, Wray et al[1] describe the relationship between the misdiagnosis rate of trait A as trait B (M_{TA}) and the number of cases diagnosed with trait A (N_{caseDA}), the genetic variance of trait A (σ_{uDA}^2) and the genetic covariance of traits A and B ($\sigma_{\mathrm{uDA,uDB}}$) as

$$N_{\mathrm{caseDA}} = (1 - M_{\mathrm{TA}})N_{\mathrm{caseTA}} + M_{\mathrm{TB}}N_{\mathrm{caseTB}}\,,$$

(Equation 4)

$$\sigma_{\mathrm{uDA}}^2 = \frac{(1 - M_{\mathrm{TA}})^2 N_{\mathrm{caseTA}}^2 \sigma_{\mathrm{uTA}}^2 + M_{\mathrm{TB}}^2 N_{\mathrm{caseTB}}^2 \sigma_{\mathrm{uTB}}^2 + 2(1 - M_{\mathrm{TA}})M_{\mathrm{TB}}N_{\mathrm{caseTA}}N_{\mathrm{caseTB}}\sigma_{\mathrm{uTA,uTB}}}{N_{\mathrm{caseDA}}^2},$$

(Equation 5)

and

$$\sigma_{\mathrm{uDA,uDB}} = \frac{[\big((1 - M_{\mathrm{TA}})(1 - M_{\mathrm{TB}}) + M_{\mathrm{TA}}M_{\mathrm{TB}}\big)N_{\mathrm{caseTA}}N_{\mathrm{caseTB}}\sigma_{\mathrm{uTA,uTB}} + (1 - M_{\mathrm{TA}})M_{\mathrm{TA}}N_{\mathrm{caseTA}}^2 + (1 - M_{\mathrm{TB}})M_{\mathrm{TB}}N_{\mathrm{caseTB}}^2\sigma_{\mathrm{uTB}}^2]}{N_{\mathrm{caseDA}}N_{\mathrm{caseDB}}},$$

(Equation 6)

where N_{caseTA} and N_{caseTB} are the total numbers of "true" cases of traits A and B respectively, M_{TB} is the misdiagnosis rate of trait B as trait A, σ_{uTA}^2 and σ_{uTB}^2 are the genetic variances of true cases of traits A and B respectively, $\sigma_{\mathrm{uTA,uTB}}$

is the genetic covariance of true cases of traits A and B and N_{caseDB} is the number of cases diagnosed with trait B. For consistency with Wray et al[1] we have used their symbols in these derivations; in the main text of this study the symbol M is used for M_{TA}, ρ_g for $\sigma_{\text{uDA,uDB}}$, N_{ALS} for N_{caseDA} and N_{SCZ} for N_{caseDB}.

Assigning ALS as trait A and schizophrenia as trait B, and reasoning that misdiagnosis of schizophrenia as ALS is highly unlikely ($M_{\text{TB}} = 0$), our specific interest is in cases where FTD-ALS (trait A) has been misdiagnosed as schizophrenia (trait B) under a true genetic correlation of 0 (ie $\sigma_{\text{uTA,uTB}} = 0$). Equations 1-3 therefore simplify to

$$N_{\text{caseDA}} = (1 - M_{\text{TA}})N_{\text{caseTA}} ,$$

(Equation 7)

$$\sigma^2_{\text{uDA}} = \frac{(1 - M_{\text{TA}})^2 N^2_{\text{caseTA}} \sigma^2_{\text{uTA}}}{N^2_{\text{caseDA}}}$$

(Equation 8)

and

$$\sigma_{\text{uDA,uDB}} = \frac{(1 - M_{\text{TA}}) M_{\text{TA}} N^2_{\text{caseTA}}}{N_{\text{caseDA}} N_{\text{caseDB}}}.$$

(Equation 9)

The assumption $M_{\text{TB}} = 0$ implies that the genetic variance estimate of diagnosed cases of trait A (σ^2_{DA}) accurately reflects the genetic variance of true cases of trait A (σ^2_{TA}); this is confirmed by substituting equation 7 into equation 8:

$$\sigma^2_{\text{DA}} = \frac{(1 - M_{\text{TA}})^2 N^2_{\text{caseTA}} \sigma^2_{\text{uTA}}}{(1 - M_{\text{TA}})^2 N^2_{\text{caseTA}}},$$

which simplifies to $\sigma^2_{\text{uDA}} = \sigma^2_{\text{uTA}}$. The misdiagnosis rate of trait A (M_{TA}) can be established by rearranging equation 7 in terms of the unknown term N_{caseTA}:

$$N_{\text{caseTA}} = \frac{N_{\text{caseDA}}}{(1 - M_{\text{TA}})},$$

and substituting this into equation 9 to give the relationship between genetic covariance ($\sigma_{\text{uDA,uDB}}$) and M_{TA}, N_{caseDA} and N_{caseDB}:

$$\sigma_{\text{uDA,uDB}} = \frac{(1 - M_{\text{TA}}) M_{\text{TA}} \frac{N^2_{\text{caseDA}}}{(1 - M_{\text{TA}})^2}}{N_{\text{caseDA}} N_{\text{caseDB}}}.$$

Simplifying this,

$$\sigma_{\text{uDA,uDB}} = \frac{M_{\text{TA}} N_{\text{caseDA}}}{(1 - M_{\text{TA}}) N_{\text{caseDB}}},$$

and expressing in terms of M_{TA} yields the relationship

$$\frac{\sigma_{\mathrm{uDA,uDB}} N_{\mathrm{caseDB}}}{N_{\mathrm{caseDA}}} = \frac{M_{\mathrm{TA}}}{(1 - M_{\mathrm{TA}})}.$$

(Equation 10)

Substituting $C = \sigma_{\mathrm{uDA,uDB}} N_{\mathrm{caseDB}} / N_{\mathrm{caseDA}}$ (the left-hand term) and solving for M_{TA} gives the relationship

$$M_{\mathrm{TA}} = \frac{C}{C + 1}.$$

(Equation 11)

Using mixed linear model GWAS association statistics for ALS and constrained intercept LD score regression, the genetic correlation (r_g) estimate for ALS and schizophrenia was 14.3% (7.05-21.6). Using the relationship $\rho_{g,l} = r_g \sqrt{h^2_{\mathrm{ALS},l} h^2_{\mathrm{SCZ},l}}$ (ref 11) where subscript l denotes liability-scale, this corresponds to a liability-scale genetic covariance of 1.88% (0.92-2.83). With GWAS case cohort sizes of $N_{\mathrm{caseDA}} = 12{,}577$ and $N_{\mathrm{caseDB}} = 34{,}241$, M_{TA} evaluates to 4.86% (2.47-7.13). Supplementary table 7 shows M_{TA} estimates for the study's other $\rho_{g,l}$ estimates.

Supplementary methods 2: Modelling expected comorbidity

Under a positive genetic correlation between two binary phenotypes, an increased number of individuals exhibiting both phenotypes would be expected. To determine the required cohort size to observe a significant excess of comorbidity, we modelled the bivariate distribution in liability for pairs of traits as $N_2(0, \Sigma)$ given the covariance matrix $\Sigma = \begin{bmatrix} 1 & \rho_{g,l} \\ \rho_{g,l} & 1 \end{bmatrix}$, where $\rho_{g,l} = r_g \sqrt{h^2_{\mathrm{ALS},l} h^2_{\mathrm{SCZ},l}}$ is the liability-scale genetic covariance. We calculated the liability thresholds t_{A} and t_{B} for traits A and B as the Normal quantiles above which proportions P_{A} and P_{B} of liability lie, corresponding to lifetime risks for traits A and B, respectively. We then calculated the expected proportion P_{AB} of cases that pass these thresholds from the bivariate cumulative distribution of liability using the algorithm of Genz et al[2] implemented in the mvtnorm package in R 3.0.2. The odds ratio for developing ALS given a diagnosis of schizophrenia is (supplementary figure 3)

$$OR_{\mathrm{ALS|SCZ}} = \frac{P_{\mathrm{AB}}/(P_{\mathrm{B}} - P_{\mathrm{AB}})}{(P_{\mathrm{A}} - P_{\mathrm{AB}})/(1 - P_{\mathrm{A}} - P_{\mathrm{B}} - P_{\mathrm{AB}})}.$$

(Equation 12)

The odds ratio for developing schizophrenia given a diagnosis of ALS is

$$OR_{\mathrm{SCZ|ALS}} = \frac{P_{\mathrm{AB}}/(P_{\mathrm{A}} - P_{\mathrm{AB}})}{(P_{\mathrm{B}} - P_{\mathrm{AB}})/(1 - P_{\mathrm{A}} - P_{\mathrm{B}} - P_{\mathrm{AB}})}.$$

(Equation 13)

Cross-multiplying, both equations become

$$OR = \frac{P_{\mathrm{AB}}(1 - P_{\mathrm{A}} - P_{\mathrm{B}} - P_{\mathrm{AB}})}{(P_{\mathrm{A}} - P_{\mathrm{AB}})(P_{\mathrm{B}} - P_{\mathrm{AB}})}.$$

(Equation 14)

Under no genetic correlation the expected proportion P_0 of individuals passing liability thresholds t_{A} and t_{B} for traits A and B is the bivariate cumulative distribution function for $N_2(0, \mathbf{I})$ at t_{A} and t_{B}, which is equivalent to $P_{\mathrm{A}}P_{\mathrm{B}}$ ($\mathbf{I}$ is the identity matrix). This was used to calculate the size of population n required to achieve 80% power ($\beta = 0.2$) to observe a significant ($\alpha = 0.05$) excess of co-occurrences of the two traits using the two-tailed binomial power equation,

$$n = P_{\mathrm{AB}}(1 - P_{\mathrm{AB}})\left(\frac{\phi^{-1}_{1-\frac{\alpha}{2}} + \phi^{-1}_{1-\beta}}{P_{\mathrm{AB}} - P_0}\right)^2,$$

(Equation 15)

where ϕ is the cumulative distribution function of the Normal distribution. Multiplying n by the lifetime risk for developing ALS (1/400) gives the required size of incident ALS cohort to observe this excess.

References

1. Wray NR, Lee SH, Kendler KS. Impact of diagnostic misclassification on estimation of genetic correlations using genome-wide genotypes. Eur J Hum Genet. 2012 Jun;20(6):668–74.

2. Genz A. Numerical Computation of Multivariate Normal Probabilities. J Comput Graph Stat. 1992 Jun;1(2):141–9.
